# Cell Transformation and the Evolution of a Field of Precancerization As It Relates to Oral Leukoplakia

**DOI:** 10.1155/2011/321750

**Published:** 2011-10-05

**Authors:** L. Feller, J. Lemmer

**Affiliations:** Department of Periodontology and Oral Medicine, University of Limpopo, Medunsa Campus, South Africa

## Abstract

Potentially malignant oral leukoplakias arise within precancerized epithelial fields consisting of cytogenetically altered keratinocytes at various stages of transformation. The evolution of a clone of keratinocytes culminating in a precancerous phenotype is a function of the number of mutagenic events, rather than the sequential order in which they occur. The altered molecular configurations of the transformed precancerous keratinocytes may confer upon them a growth advantage in relation to the unaltered neighbouring keratinocytes. Replicative clonal expansion of these keratinocytes results in the progressive replacement of the surrounding normal keratinocytes by the fitter clone or clones of altered cells. The precancerized oral epithelial field may have a clinically normal appearance and microscopically may be normal or may show dysplasia. Oral leukoplakias arising within a precancerized epithelial field in which the keratinocytes show DNA aneuploidy or loss of heterozygosity at certain specific chromosomal loci have the potential to progress to carcinoma. The pathogenic mechanisms that drive the carcinomatous transformation of oral leukoplakias, in which cytogenetic alterations in the keratinocytes cannot be detected, are unknown.

## 1. Introduction

The genome can sustain a number of alterations to its DNA without any apparent cellular functional disability, but any cell that has sustained a critical number of DNA alterations, such that one or two additional alterations are likely to result in malignant change, is regarded as being transformed. 

It is evident that potentially malignant oral leukoplakias arise within fields of precancerization consisting of cytogenetically altered keratinocytes [1, 2]. A field of precancerization in the oral cavity can be defined as an area of clinically normal-looking epithelium which is either microscopically normal or shows dysplasia, but in which some keratinocytes have undergone cytogenetic alterations [1–8]. The process of progressive cytogenetic alteration (transformation) can confer upon the keratinocytes in such an epithelial field a growth advantage in relation to the normal surrounding keratinocytes [9] so that within an apparently clinically normal stretch of oral mucosa there can be a pathobiological continuum from normal epithelium to precancerized epithelium where carcinoma can arise [7].

## 2. An Oral Field of Precancerization

A field of precancerized oral epithelium comprises a large number of cytogenetically altered keratinocytes at various stages of transformation. It is not yet clear how many genetic events are necessary to establish cellular transformation, and not all the molecular alterations associated with field precancerization of normal-looking oral epithelium have been characterized [1].

The initial transformational events occur in progenitor cells in the basal and/or parabasal cell layers of the oral epithelium [10, 11]. These progenitor cells give rise to cells that maintain the integrity of the basal and parabasal cell layers and to amplifying cells that divide frequently, producing daughter cells that enter the maturation process, and gradually progress to the surface of the epithelium [12].

The initial cytogenetic events leading to cell transformation are most probably characterized by molecular and subsequent functional alterations to genes maintaining the cell's genomic stability. Such alterations precipitate sequential cytogenetic lesions driving the genetic evolution of the initially transformed progenitor cell towards a cell with a complete set of genetic alterations of a malignant phenotype [13–17]. In this regard, the evolution of a cancerous keratinocyte is the outcome of a sufficient number of accumulated cytogenetic alterations rather than of the sequential order in which they occur [15, 18–20].

The cytogenetic alterations in precancerous keratinocytes reflect a noncritical accumulation of molecular events, and those seen in cancerous keratinocytes are the outcome of a further critical accumulation of transforming events. The exact sequence of the cytogenetic molecular events culminating in carcinogenesis can vary [14, 21].

Most of the cytogenetic and transcriptional alterations occur early in the continuum from normality, to precancerization, to carcinoma. It is probable that a greater number of such alterations are required for the transformation of a normal to a precancerous keratinocyte, than for the transformation of a precancerous keratinocyte to a keratinocyte expressing a full cancerous phenotype [22].

The number of sequential cytogenetic alterations necessary for a progenitor cell to become a cancer cell is imponderable, but is estimated to be between 3 and 12. The exact number of these singular genetic events is a function of several factors: loss of DNA repair mechanisms, dysregulation of signal transduction pathways, and blocking of mechanisms leading to apoptosis [23, 24], which together will selectively confer an advantage upon the transformed cells with regard to competitive fitness and growth in relation to the surrounding normal cells [9]. This cytogenetic evolution will promote the clonal expansion of the transformed cell population and their subsequent clonal divergence to form either a single precancerized epithelial field or multiple fields that may be contiguous with or separate from each other, at the expense of the surrounding normal epithelium [1].

A field of precancerization may comprise a monoclonal cell population originating from one transformed progenitor basal cell that underwent clonal expansion, and spread laterally. The molecular profile of the transformed keratinocytes in such a field may be identical if they are all part of a monoclone. On the other hand, the molecular profile of the transformed keratinocytes may be similar to but not identical with one another (clonally related) if they arose from subclones related by descent from the original monoclone, but each subclone having experienced episodes of different mutations [1, 25–29].

However, a field of precancerization may also consist of a polyclonal cell population, several transformed progenitor basal cells having undergone independent clonal expansion with subsequent independent clonal divergence. The molecular profiles of the transformed keratinocytes in such a field will be dissimilar, but might share some cytogenetic alterations, as the polyclonal cells will have been exposed to identical carcinogens and to the same microenvironment in the mouth. Thus, the keratinocytes of a unique precancerous oral leukoplakia or of a unique oral squamous cell carcinoma can show molecular heterogeneity with similar or dissimilar cytogenetic alterations [1, 25–29]. 

The precancerous field may be clinically normal, whether or not there is epithelial dysplasia. The presence of a field of precancerization may be disclosed by the development of a leukoplakia or of an erythroplakia, or the appearance of squamous cell carcinoma will make the nature of the previously unrecognized field of precancerization become all too apparent [1].

The cytogenetic events culminating in the development of a field of precancerization can be either spontaneous or microenvironmentally induced, or extraneously imposed, but it is possible that some ectodermal stem cells destined to become progenitor cells in the oral epithelium can actually undergo genetic and/or epigenetic alterations during organogenesis, giving rise to cytogenetically altered epithelial progenitor cells. If during maturation these developmentally altered keratinocytes are exposed to further transforming molecular events, their clonal expansion can culminate in the formation of a precancerized field [1, 10, 11, 25, 30].

It is possible that dysregulation of gene expression in the fibroblasts of the lamina propria, with consequent production and release of aberrant mediators, may impose upon the overlying keratinocytes that have already undergone initial transformation from whatever cause, dysregulatory inductive signals contributing to the development of the precancerized field [1, 31–33].

## 3. The Failure of the Studies of Potentially Malignant Lesions to Contribute to Knowledge of Potentially Malignant Lesions of the Mouth

There are a number of potentially malignant lesions of the upper aerodigestive tract in which the oral cavity, nasopharynx, oropharynx, hypopharynx, and the larynx, are grouped together as if premalignancies of these sites constitute a homogeneous group. Potentially malignant lesions from these various sites are, however, site specific, so such studies are of limited value in isolating molecular and cytogenetic markers of carcinomatous transformation of oral leukoplakia [1].

Furthermore, although the potentially malignant lesions of the upper aerodigestive tract have common histological features of epithelial dysplasia or hyperplasia, they certainly do not have similar clinical features or natural courses. In addition, in several studies, oral epithelial dysplasia and hyperplasia have been chosen as the inclusion criteria for categorising oral lesions as being potentially malignant, without differentiating the clinical entities. Moreover, certain reactive and inflammatory lesions of the oral mucosa exhibiting epithelial hyperplasia or low-grade dysplasia have been erroneously included in some of the studies although these lesions will never undergo carcinomatous transformation [1].

## 4. Molecular Markers of Oral Leukoplakia and Its Progression to Squamous Cell Carcinoma

The development of precancerous oral leukoplakia and its carcinomatous transformation can be brought about by dysregulation of the mechanisms controlling the cell cycle, DNA repair, and apoptosis, by activation of proto-oncogenes, by loss of chromosomal regions containing candidate tumour-suppressor genes as a result of loss of heterozygosity (LoH), and by alterations to genomic DNA and to mitochondrial DNA [23–25, 34, 35]. While these molecular and cytogenetic markers may not be reliable predictors of carcinomatous transformation [1, 34, 36–38], there is a good deal of evidence that LoH at specific chromosomal arms, and aneuploidy, are indeed associated with increased frequency of carcinomatous transformation of oral leukoplakia [35, 39–43]. However, irrespective of one or more cytogenetic or molecular changes to the keratinocytes of oral leukoplakia, progression to carcinoma may never occur [1]. 

LoH at the chromosome arms 3p and 9p occur frequently in keratinocytes of precancerous epithelial lesions of the upper aerodigestive tract. This pattern of LoH can be associated with carcinomatous transformation of these lesions [18, 35, 40, 44, 45]. In one study, precancerous epithelial lesions with keratinocytes having LoH limited to 3p and/or 9p had a 3.8-fold higher risk of carcinomatous transformation compared to precancerous lesions without LoH either at 3p or at 9p, while those lesions with keratinocytes carrying LoH at 3p and/or 9p, together with additional losses at any of the chromosomes 4q, 8p, 11q, and 17p had a 33-fold increased risk of carcinomatous transformation, compared to precancerous lesions with keratinocytes retaining these chromosomal arms, and also progressed to carcinoma more rapidly, although unpredictably [35, 41].

For unknown reasons, keratinocytes of dysplastic oral leukoplakias on the floor of the mouth, on the ventrolateral surface of the tongue, and at the maxillary retromolar/soft palate region have LoH more frequently than do the keratinocytes of dysplastic leukoplakias affecting other regions of the oral mucosa. This may be one of the factors putting leukoplakias at the three sites of the oral mucosa mentioned, at higher risk of carcinomatous transformation than leukoplakias at other lower-risk sites [41].

Notwithstanding, the increased risk of carcinomatous transformation associated with LoH in precancerous epithelial lesions, not all oral leukoplakias with keratinocytic LoH, will progress to carcinoma, and as some oral leukoplakias without keratinocytic LoH will indeed progress to carcinoma, LoH is certainly not an infallible predictor of carcinomatous transformation [1]. 

It seems that the presence of DNA aneuploidy in keratinocytes of oral leukoplakia may be a valuable molecular marker of carcinomatous transformation of oral leukoplakia and an indicator of poor prognosis. The frequency of carcinomatous transformation of dysplastic oral leukoplakia is much greater for those leukoplakias with keratinocytes showing DNA aneuploidy than for those with keratinocytes with normal (diploid) DNA content, and subjects with oral carcinoma developing from dysplastic leukoplakia with keratinocytes manifesting aneuploidy have a lower rate of survival compared to subjects with oral squamous cell carcinoma evolving from dysplastic leukoplakia with keratinocytes with diploid DNA content [43, 46].

## 5. Field Precancerization and Oral Leukoplakia

The existence of a field or fields of precancerization can explain why oral leukoplakia may manifest at single or at multiple sites, synchronously and/or metachronously and may recur at the same site from which it was previously apparently successfully excised [1]. 

Additional cytogenetic alterations superimposed upon transformed precancerous keratinocytes either within a leukoplakia or within a clinically normal-looking zone of an epithelial field of precancerization may drive the process of cancerization. This explains why sometimes an oral leukoplakia on microscopical examination is found to have already become squamous cell carcinoma, and sometimes squamous cell carcinoma arises apparently *de novo* either contiguous to or separated from existing leukoplakia. If a substantial portion of a field of precancerization arises from a monoclone of cytogenetically transformed keratinocytes, then any leukoplakias, or subsequently developing carcinomas arising within that field either contiguously or separately from each other, will be cytogenetically very similar or identical. On the other hand, if a field of precancerization comprises a number of clones of cytogenetically dissimilarly transformed keratinocytes, then leukoplakias or carcinomas arising within this field (if multiple lesions should occur) will be cytogenetically dissimilar [1, 30].

The transformed keratinocytes in the precancerized field from which a leukoplakia had apparently been completely excised may give rise to “recurrence” of another leukoplakia from cytogenetically related subclones of transformed keratinocytes adjacent to the surgical excision or from adjacent cytogenetically unrelated clones [45].

The time of progression of oral leukoplakias with keratinocytes with molecular profiles associated with carcinomatous transformation, to squamous cell carcinoma if it occurs, unpredictably and inexplicably varies from six months to eight years [35, 41]. However, it may sometimes take more than 30 years for oral leukoplakia to progress to squamous cell carcinoma [47], and the average period for carcinomatous transformation ranges from 5 to 8 years [47, 48]. Neither the rate of progression of oral leukoplakia to carcinoma nor the mechanisms bringing about the regression of oral leukoplakia, can be explained in terms of the cytogenetic characteristics of the precancerous field. 

The mechanisms that bring about regression of oral leukoplakia in which the keratinocytes harbour cytogenetic changes associated with early transformational events are obscure. However, it is possible that a continuous accumulation of cytogenetic alterations may give rise to a precancerous clone of cells that is inferior rather than superior in its competitive growth and fitness resulting in its replacement by, rather than in its replacing, the normal neighbouring keratinocytes [1]. Alternatively, or additionally, the accumulated cytogenetic alterations may result in failure of DNA replication and cell viability leading to the extinction of the precancerous clone [14].

## 6. Summary

Despite advances in molecular genetic studies, there are no molecular markers that are reliable predictors of progression of oral leukoplakia to carcinoma. Although some oral leukoplakias arise within fields of precancerized epithelium where initial cytogenetic alterations have already occurred, their transformed keratinocytes may or may not acquire complete malignant phenotypes.
